# Incidence trend analysis of human brucellosis in the context of human development: an ecological study using joinpoint regression in Iran

**DOI:** 10.1186/s12889-025-25304-5

**Published:** 2025-11-17

**Authors:** Marziyeh Hamyali-Ainvand, Mohammad Ebrahimi

**Affiliations:** Student Research Committee, Shiraz University of Medical Sciences, Shiraz, Iran

**Keywords:** Human brucellosis, HDI, Time trend analysis, Iran, Joinpoint regression, Ecological study

## Abstract

**Background:**

Human brucellosis (HB) remains a significant public health concern in Iran, where its burden may be shaped by socio-developmental factors. This ecological time trend analysis explores long-term patterns in HB incidence in the context of human development.

**Methods:**

We conducted Joinpoint regression analysis to assess temporal trends in HB incidence from 2000 to 2023 in Iran. The Human Development Index (HDI) was included as a predictor to examine associations using Spearman correlation and linear regression on log-transformed HDI. Residual diagnostics and robust regression techniques were applied.

**Results:**

The overall annual percentage change (AAPC) in HB incidence showed a slight but statistically significant upward trend (+ 0.45%; *p* = 0.023). Joinpoint regression identified four statistically significant changes in trend. A moderate negative correlation was observed between HB incidence and HDI (Spearman’s ρ = − 0.54). Linear regression indicated an inverse association between log(HDI) and HB incidence (β = − 65.96, *p* = 0.044), though the explained variance was limited (R² = 0.226).

**Conclusion:**

The findings suggest that higher levels of human development may be associated with reduced HB incidence at the national level. However, this association is likely influenced by unmeasured factors such as access to veterinary care, cultural practices, and surveillance quality. Caution is warranted in interpreting these findings due to the ecological study design and potential underreporting.

**Supplementary Information:**

The online version contains supplementary material available at 10.1186/s12889-025-25304-5.

## Introduction

Brucellosis, a significant zoonotic disease of bacterial etiology, primarily impacts both livestock populations and human health globally [1, 2]. The disease is caused by several species within the genus Brucella, notably B. abortus, B. melitensis, and B. suis, and transmission to humans typically occurs through the consumption of unpasteurized dairy products or direct contact with infected animal tissues [3–5]. Clinical manifestations of human brucellosis (HB) include fever, sweating, fatigue, and in chronic cases, arthritis and neuropathy. Individuals with occupational exposure to livestock, such as farmers, slaughterhouse workers, and veterinarians, are at an elevated risk of contracting the disease [5–7]. Iran, a country in the Eastern Mediterranean region with one of the highest incidences of brucellosis, has reported variable trends in its occurrence in recent years [8, 9]. As an endemic disease in Iran, brucellosis remains a significant public health concern, particularly in rural areas where close contact with livestock is common [10–12]. This disease is prevalent in over 170 countries worldwide, and developing nations such as Iran continue to bear a significant burden. The reported incidence rate in Iran ranges from less than 0.03 to over 200 cases per 100,000 population [10, 13].

The Human Development Index (HDI) is a composite statistic developed by the United Nations Development Programme to capture the multidimensional aspects of human progress, integrating indicators of health (life expectancy), education (mean and expected years of schooling), and income (GNI per capita) [14–16]. While HDI has been widely employed in cross-national health studies, its relationship with infectious disease incidence at the national level over time remains underexplored, particularly in regions affected by structural inequalities, economic sanctions, and fluctuating health investment [16–18]. Examining disease trends within the framework of the HDI can elucidate the relationship between socio-economic factors and health, thereby aiding in the design of targeted interventions to promote health equity [17, 18].

Recent studies have highlighted the relevance of socio-developmental determinants in shaping the epidemiology of zoonoses. Higher levels of human development may correlate with improved health systems, better surveillance capacity, and reduced occupational exposure to livestock—factors that could potentially mitigate HB incidence. The HDI may influence disease incidence trends by shaping healthcare capacity and reflecting underlying socio-economic shifts that affect national health outcomes [19–22]. Given that Iran’s HDI has undergone both periods of advancement and stagnation over the past two decades—affected by economic sanctions, policy fluctuations, and uneven regional development—analyzing its association with diseases such as human brucellosis (HB) can help clarify how national and subnational socio-economic dynamics influence infectious disease patterns in the country [23–25]. However, the ecological nature of such analyses introduces substantial limitations, including the risk of ecological fallacy—drawing individual-level inferences from population-level data. These limitations underscore the importance of cautious interpretation and clear articulation of study boundaries.

This study applies Joinpoint regression and regression modeling to investigate temporal trends in HB incidence in Iran over a 23-year period (2000–2023) and its association with HDI. The objective is to analyze the temporal trend of HB in Iran and to evaluate its association with the HDI within an ecological framework using advanced statistical models, including Joinpoint regression and correlation analysis.

While the study adopts a conceptual One Health perspective, integrating socioeconomic dimensions of zoonotic disease control, no animal or environmental datasets were available for inclusion. Our aim is to contribute to understanding how macro-structural determinants such as HDI may align with long-term shifts in HB incidence, generating hypotheses for more granular, multilevel analyses in future research and providing valuable insights into the role of development indicators in shaping public health outcomes and informing evidence-based health policy and disease control strategies.

## Methods

### Study design and data sources

This study employed an ecological design to examine the time trend of human brucellosis (HB) incidence in Iran from 2000 to 2023 and its association with the Human Development Index (HDI). Annual data on HB incidence were obtained from official sources within the Iranian Ministry of Health [26]. Due to the absence of disaggregated data, age- or sex-standardized rates could not be computed.

Annual population data for the country during the study period were obtained from the Population Division of the United Nations Department of Economic and Social Affairs (UNDESA), a recognized and reliable source for demographic statistics, due to its more precise population estimates [27]. National-level HDI data were extracted from the Global Human Development Reports issued by the United Nations Development Programme (UNDP), available through 2022 [24]. 

### Statistical analysis

The annual crude incidence rate was calculated by dividing the number of reported cases by the population of the corresponding year and multiplying by 100,000.

Trend analysis was conducted using the Joinpoint Regression Program (version 5.4.0), developed by the U.S. National Cancer Institute. This method fits segmented regression lines to log-transformed incidence data to detect statistically significant changes in trend patterns. In this model, the dependent variable was the annual crude incidence rate of HB, while the independent variable was calendar year, treated as a continuous predictor. The maximum number of joinpoints was set to four, consistent with the number of annual observations and established methodological recommendations. A sensitivity analysis with fewer joinpoints (reduced-k) produced similar direction and magnitude of AAPC estimates, confirming model robustness. Model selection was guided by permutation tests and the Bayesian Information Criterion (BIC). Annual Percentage Changes (APCs) and the Average Annual Percentage Change (AAPC) were computed, along with their 95% confidence intervals (CIs), using 3001 bootstrap resamples, as recommended by the software documentation [28].

To examine the association between HDI and HB incidence, linear regression analysis was applied. Given the right-skewed distribution of HDI values and the presence of multiplicative variance, a logarithmic transformation was performed on HDI prior to modeling. The regression model evaluated the relationship between crude HB incidence and log-transformed HDI. Normality of residuals was assessed using the Shapiro–Wilk test and Q–Q plots. To ensure robustness against deviations from normality or the presence of outliers, we employed a robust regression model with bootstrapped standard errors based on 5,000 resamples. The potential for residual autocorrelation was tested using the Durbin–Watson statistic. A quadratic term for log(HDI) was also tested to explore the potential for a nonlinear relationship between socioeconomic development and disease incidence.

The normality of HDI and HB crude incidence rate (CIR) distributions was assessed using Shapiro–Wilk and Kolmogorov–Smirnov tests, which indicated non-normality. As a result, HDI values were log-transformed, and Spearman’s rank correlation was applied to assess the monotonic association between HDI and HB incidence for the period 2000–2022. Due to the mismatch in data availability—HDI up to 2022 and HB incidence through 2023—correlation and regression analyses were restricted to 2000–2022, while data from 2023 were included solely in trend analyses.

All statistical analyses were conducted using IBM SPSS Statistics version 26, and a p-value of less than 0.05 was considered statistically significant [29].

## Results

### HB time trend

In the present study, the average annual crude incidence rate (CIR) of HB was 23.96 per 100,000 population (23.96 ± 5.75), with values ranging from 16.2 to 37.2 during the study period. The lowest incidence rate was observed in 2010 (16.2 per 100,000 population), and the highest rate was recorded in 2006 (37.2 per 100,000 population) (Table 1).

**Table 1:** Distribution of the population, number of human brucellosis cases, human brucellosis crude incidence rate (CIR) in Iran for the period 2000–2023

Year	Iran Population	HB Number	HB CIR (per 100000)
2000	65,544,383	15,022	22.9
2001	66,674,851	15,188	22.8
2002	67,327,117	16,471	24.5
2003	67,954,699	17,765	26.1
2004	69,061,674	21,530	31.2
2005	70,182,594	25,755	36.7
2006	71,275,760	26,549	37.2
2007	72,319,418	23,558	32.6
2008	73,318,394	21,109	28.8
2009	74,322,685	17,501	23.5
2010	75,373,855	12,248	16.2
2011	76,342,971	12,891	16.9
2012	77,324,451	14,208	18.4
2013	78,458,928	16,019	20.4
2014	79,961,672	19,094	23.9
2015	81,790,841	20,117	24.6
2016	83,306,231	18,518	22.2
2017	84,505,076	15,358	18.2
2018	85,617,562	15,640	18.3
2019	86,564,202	16,632	19.2
2020	87,290,193	19,455	22.3
2021	87,923,432	17,807	20.3
2022	88,550,570	20,406	23.0
2023	89,172,767	22,073	24.8

**Fig. 1:**
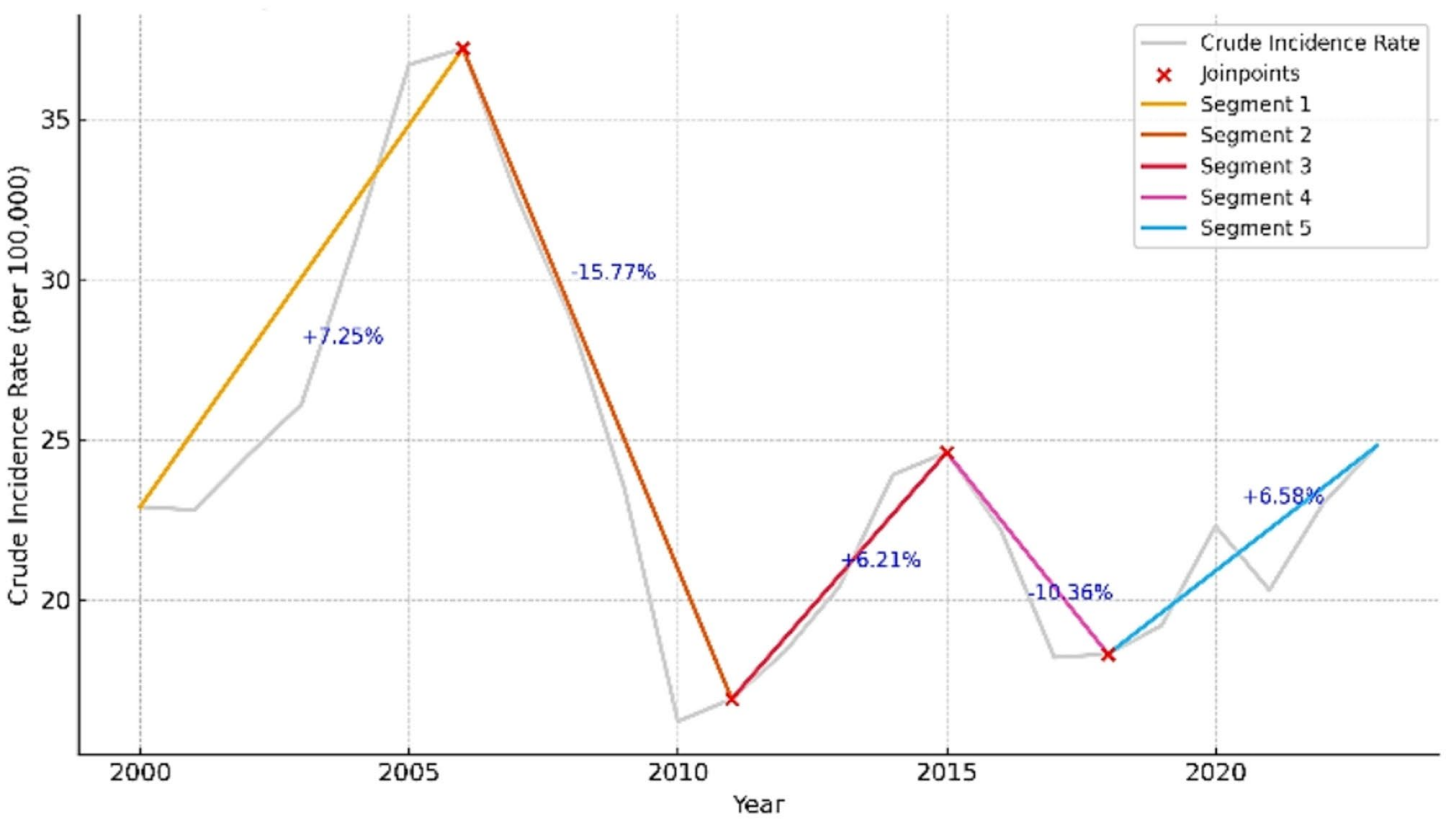
Segmented trend in the annual crude incidence rate of human brucellosis in Iran (2000–2023) based on Joinpoint regression. The model identified four statistically significant joinpoints using the Bayesian Information Criterion (BIC), with each segment annotated by its corresponding Annual Percent Change (APC). These inflection points may correspond to key historical or public health events affecting disease surveillance, policy, or reporting systems

Between 2000 and 2023, the incidence rate of human brucellosis (HB) in Iran demonstrated fluctuating trends. The Joinpoint regression analysis identified four significant inflection points over this period, as illustrated in Fig. 1. These joinpoints delineated five distinct segments, each with its own direction and magnitude of change in HB incidence. Joinpoint analysis results are detailed in Table 2. Each segment identified by a joinpoint is accompanied by its corresponding Annual Percentage Change (APC) and 95% confidence interval. The Average Annual Percentage Change (AAPC) across the full study period was estimated at + 0.45% (95% CI: +0.11% to + 1.36%, *p* = 0.023), indicating a statistically significant but modest upward trend in HB incidence. Confidence intervals and p-values for Annual Percent Changes (APCs) were estimated using 3,001 bootstrap resamples, as recommended by the Joinpoint Regression Program.

**Table 2:** Trends analysis of human brucellosis incidence in Iran using joinpoint regression model (2000–2023)

Time Interval	Joinpoint Year (95% CI)	APC (%)	95% CI	*p*-value
2000–2006	Joinpoint 1: 2006 (2005–2007)	+ 7.25	+ 5.01 to + 9.34	< 0.001
2006–2011	Joinpoint 2: 2011 (2010–2012)	–15.77	–20.38 to − 12.19	< 0.001
2011–2015	Joinpoint 3: 2015 (2014–2015)	+ 6.21	–2.50 to + 13.45	0.088
2015–2018	Joinpoint 4: 2018 (2018–2020)	–10.36	–14.67 to − 3.80	< 0.001
2018–2023	—	+ 6.58	+ 5.33 to + 12.30	0.001
2000–2023	—	AAPC = + 0.45	+ 0.11 to + 1.36	0.023

### HDI trend

**Fig. 2:**
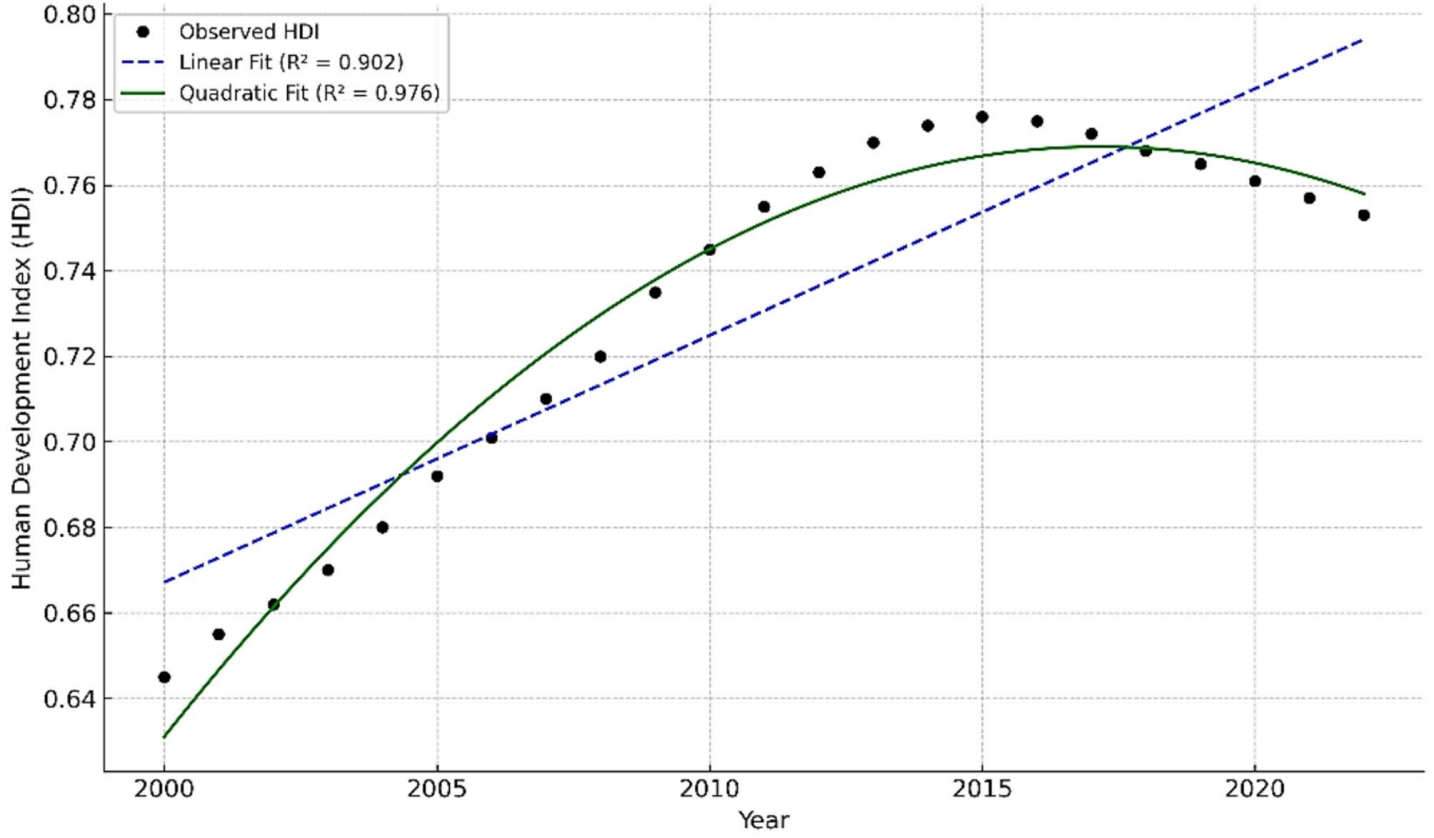
Temporal trend of the Human Development Index (HDI) in Iran from 2000 to 2022, modeled using both linear and quadratic regression. The quadratic model demonstrated superior fit (R² = 0.976) compared to the linear model (R² = 0.902), reflecting potential

The trend of the HDI in Iran during the study period (2000–2022) was characterized by an initial increase between 2000 and approximately [e.g., 2010], followed by a deceleration and a subsequent decline beginning in the mid-2010s. A quadratic regression model demonstrated a strong goodness-of-fit (R² = 0.976) (Fig. 2).

### Correlation

Assessment of data normality using the Kolmogorov–Smirnov (*p* = 0.003) and Shapiro–Wilk (*p* = 0.006) tests revealed that HDI was not normally distributed (Table 3). Consequently, to evaluate the relationship between HDI and the incidence of human brucellosis, Spearman’s rank-order correlation was employed. The analysis demonstrated a statistically significant inverse correlation between these two variables (Spearman’s rho = −0.543, *p* = 0.007) (Table 4).

**Table 3:** Tests of normality for the human development index (HDI) using Kolmogorov-Smirnov and Shapiro-Wilk tests

Tests of Normality						
	Kolmogorov-Smirnov^a^			Shapiro-Wilk		
	Statistic	df	Sig.	Statistic	df	Sig.
HDI	0.228	23	0.003	0.870	23	0.006

^a^Lilliefors Significance Correction

**Table 4:** Spearman’s correlation between human development index (HDI) and human brucellosis incidence rate (HB CIR)

Correlations				
			HDI	HB CIR
Spearman’s rho	HDI	Correlation Coefficient	1.000	− 0.543^**^
		Sig. (2-tailed)	.	0.007
		N	23	23
	HB CIR	Correlation Coefficient	**− 0.543** ^**^	1.000
		Sig. (2-tailed)	**0.007**	.
		N	23	23

**Values in bold indicate statistical significance at the 0.01 level (2-tailed).

### Regression analysis

**Fig. 3:**
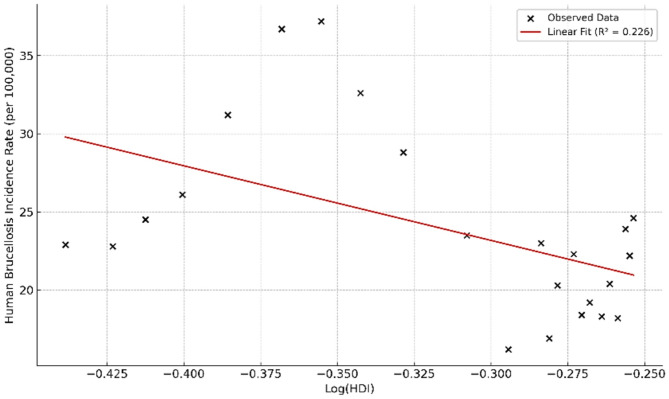
Quadratic regression illustrating the non-linear association between the log-transformed Human Development Index (HDI) and the crude incidence rate of human brucellosis in Iran (2000–2022). The estimated coefficient (β = − 65.959; *p* = 0.044) indicates a statistically significant inverse relationship, though ecological interpretation requires caution. Bootstrap-based confidence intervals (5,000 resamples) were applied due to mild non-normality in residuals

To improve linearity/homoscedasticity and residual diagnostics, a logarithmic transformation was applied. Graphical examination using a scatter plot (Fig. 3) indicated a nonlinear relationship between the log-transformed HDI and the crude incidence rate of human brucellosis, with a quadratic model showing a coefficient of determination (R²) of 0.437. A simple linear regression model was fitted to assess the linear association between the log-transformed HDI and human brucellosis incidence. The results revealed a statistically significant inverse association (β = − 65.959; 95% CI: − 147.872 to − 28.340; *p* = 0.044), with the estimated regression equation: HB CIR = 5.397–65.959(Log HDI). The coefficient of determination (R² = 0.226) indicated that approximately 22.6% of the variance in brucellosis incidence could be statistically explained by variations in the log-transformed HDI. Given the violation of the normality assumption for the independent variable (Log HDI) (Shapiro–Wilk *p* = 0.006; Kolmogorov–Smirnov *p* = 0.003), bootstrap analysis with 5,000 resamples was conducted to validate the model estimates and improve their precision (Table 5).

**Table 5:** Simple linear regression of Log-Transformed human development index on human brucellosis incidence in Iran (2000–2023)

Variable	B (Coefficient)	Standard Error (SE)	Standardized Beta	t		Significance (*p*)	95% CI (Bootstrap)
Constant	5.397	7.628	–		0.714	0.424	(−15.517, 15.536)
Log HDI	−65.959	29.442	−0.475		−2.24	0.044	(−147.872, −28.340)

Note. Standardized beta reported without CI; bootstrap CIs apply to unstandardized coefficients

The Durbin–Watson statistic was 1.89, indicating no evidence of residual autocorrelation

## Discussion

This ecological study examined the national-level temporal trends of human brucellosis (HB) incidence and its association with the Human Development Index (HDI) in Iran between 2000 and 2023. The key findings were as follows: (1) the incidence of HB showed considerable fluctuations over the study period, with both increasing and decreasing phases identified through Joinpoint regression analysis; (2) a statistically significant inverse association was observed between HB incidence and HDI, suggesting that improvements in socio-developmental conditions may contribute to reductions in disease burden; and (3) the coefficient of determination (R² = 0.226) was relatively low in the linear model, indicating that HDI alone explains only a modest proportion of the variation in HB incidence across time.

It is important to reiterate that, due to the ecological design, the observed associations do not imply causality at the individual level. While these findings provide valuable insights into potential links between development and zoonotic disease patterns, caution is warranted in interpreting the results due to the ecological nature of the study, which precludes individual-level inference and is susceptible to ecological fallacy.

Notably, because HDI data were only available up to 2022, regression and correlation analyses were restricted to the 2000–2022 period to ensure temporal alignment of variables. The HB incidence data for 2023 were included exclusively in the Joinpoint trend analysis, which does not require covariates. This decision was made to preserve the validity of the inferential models, and the resulting limitation is explicitly acknowledged in the discussion of methodological constraints.

### HB time trend

The findings of this study demonstrate substantial temporal variability in the crude incidence rate (CIR) of human brucellosis (HB) in Iran between 2000 and 2023, with an overall average of 23.96 cases per 100,000 population (SD ± 5.75) and annual values ranging from 16.2 to 37.2. These trends are consistent with previous national reports and studies conducted in Iran [30, 31]. The fluctuations observed may stem from multiple overlapping drivers, including climatic extremes (e.g., droughts and floods), economic instability, reduced public health funding, and evolving agricultural practices. Additionally, alterations in diagnostic protocols, improvements in surveillance coverage, shifts in Brucella species, antibiotic resistance, or policy changes may have contributed to these variations [32–38]. Underreporting due to limited diagnostic access—particularly in rural and underserved areas—and inconsistent case definitions must also be considered as important contributors [11, 39]. Notably, some joinpoints (e.g., in 2006, 2011, 2015, and 2018) may reflect transitions in surveillance systems rather than actual shifts in disease burden.

Joinpoint regression analysis identified four statistically significant change points, suggesting that the HB incidence trend was not linear or stable across the entire period. Although the overall Average Annual Percent Change (AAPC) was modest at + 0.45% (95% CI: 0.11 to 1.36, *p* = 0.023), the segmented trends showed considerable variation. For instance, the marked increase during 2005–2006 (+ 10.32%) may indicate localized outbreaks or reporting improvements, whereas the sharp decline between 2006 and 2011 (–15.77%) could be linked to intensified control efforts or systemic changes in data recording. Wide confidence intervals during 2011–2015 suggest data quality issues or external confounders. The decline in 2015–2018 (–10.36%) and the subsequent increase from 2018 to 2020 (+ 6.58%) further underscore the influence of fluctuating public health priorities and environmental conditions. This finding aligns with results from similar studies in the Mediterranean and Middle East regions, which report the highest incidence rates globally and where brucellosis occurrence is often linked to climatic variations and shifts in livestock farming practices [40–42]. These complex trends reinforce the need for stable, adaptive surveillance systems and emphasize the interconnected nature of ecological and socioeconomic factors in shaping disease dynamics.

Human brucellosis in Iran has been shown to follow a clear seasonal pattern, with most cases occurring in spring and early summer (approximately March–June), coinciding with livestock birthing and milking seasons when human contact with animals and their products increases [43–46]. Although regional variation exists—for example, peaks reported in winter months in southern provinces—the overarching pattern suggests that reproductive cycles of livestock and seasonal husbandry practices strongly influence transmission dynamics [47, 48]. In addition to seasonality, livestock vaccination campaigns represent a major driver of long-term fluctuations in incidence. In Iran, vaccination of sheep and goats with the Rev.1 vaccine and cattle with the RB51 vaccine is carried out on an annual or biannual basis as part of national control programs [30, 49]. Evidence indicates that when coverage is adequate, these campaigns substantially reduce prevalence in animal reservoirs and thereby lower human incidence. However, challenges such as incomplete coverage, logistical barriers in remote areas, and insufficient monitoring systems limit their sustained effectiveness [50, 51].

While seasonality is well documented, evidence for multi-annual epidemic cycles of human brucellosis remains less conclusive. Theoretically, such cycles could arise from oscillations in livestock susceptibility, variation in vaccination coverage, or environmental and management factors. Nonetheless, robust confirmation of multi-year cycles requires high-resolution longitudinal datasets and the application of time-series models such as ARIMA or SARIMA [52, 53]. Given the annual and national aggregation of data in the present study, we could not directly assess these cyclical dynamics. Future research incorporating monthly or regional data would allow more detailed evaluation of seasonal peaks and the influence of vaccination campaigns on epidemic periodicity.

Joinpoint regression was selected over traditional time-series models due to its capacity to detect significant inflection points in long-term, non-seasonal trends using annual data.

The selection of the optimal model with four joinpoints based on the Weighted Bayesian Information Criterion (BIC), along with confidence interval estimation using the empirical quantile method, enhances the credibility of these findings and highlights the value of advanced statistical tools in epidemiological analysis.

### HDI trend

The Human Development Index (HDI) in Iran showed a marked upward trajectory from 2000 to the mid-2010s, followed by a stagnation and eventual decline in more recent years. A quadratic regression model (R² = 0.976) better captured this nonlinear trend compared to a linear approach. The observed HDI curve likely reflects broader structural developments in Iran, such as improvements in health and education access during early years, followed by economic hardship, intensified sanctions, and public sector budget constraints post-2018. These socioeconomic changes may have indirectly influenced disease control efforts, access to veterinary care, and public health infrastructure. However, national-level HDI aggregates may not fully capture within-country inequalities, making it important to interpret these trends cautiously.

### Correlation and regression analysis

The statistical analyses revealed a moderate, inverse correlation between HDI and HB incidence (Spearman’s rho = − 0.543, *p* = 0.007), and a significant negative association in the linear regression model (β = − 65.959, *p* = 0.044). The findings from the regression analysis revealed that as the level of human development improved—reflecting advancements in education, income, and health conditions—the incidence of human brucellosis decreased. This is consistent with prior evidence showing that socioeconomic development can reduce zoonotic disease risks by limiting exposure to infected animals, improving hygiene, and enhancing access to care [54–57].

While these findings align with literature linking higher development levels to lower infectious disease burdens [57–59], the linear model explained only a limited portion of the variance (R² = 0.226), suggesting that HDI, though informative, is not a standalone predictor of HB incidence. The estimated determinant role of HDI in disease variations was approximately 23%, indicating that a substantial portion of the fluctuations in brucellosis incidence may be attributable to other intervening factors such as climatic conditions, livestock transportation, healthcare system structure, control policies, and dietary culture. Other unmeasured variables—such as livestock vaccination coverage, food safety regulations, veterinary capacity, and environmental changes—likely contribute to disease trends. These complexities highlight the importance of considering HDI as one of several contextual indicators in zoonotic disease modeling. Future analyses grounded in a One Health perspective may better capture the complex interactions between human, animal, and environmental health domains.

The improved fit of a quadratic model in the HDI trend analysis (R² = 0.976) raises the possibility of a nonlinear, perhaps inverse-U shaped association between development and HB incidence. During early stages of development, greater urban–rural interaction, agricultural expansion, and insufficient veterinary oversight may lead to increased disease exposure. However, after surpassing a certain threshold, continued improvements in infrastructure, sanitation, veterinary surveillance, and public awareness may result in a decline in disease rates. This hypothesized threshold effect, while conceptually plausible, warrants further investigation using more granular and longitudinal data sources.

Beyond statistical modeling, the visual comparison between HDI trends and HB incidence—particularly during the periods of 2006–2011 and 2018–2022—supports the inverse association hypothesis. Due to the non-normal distribution of HDI values, a log transformation was applied to meet model assumptions. Although the normality assumption for the independent variable was initially violated, the application of bootstrapped regression (5,000 resamples) validated the robustness of the parameter estimates and increased confidence in the findings. Residual diagnostics indicated no significant autocorrelation, supporting the model’s statistical reliability.

However, the analysis was limited by the lack of HDI data for 2023. To avoid temporal mismatch, we restricted regression and correlation models to the 2000–2022 period and retained 2023 data only for trend analysis (Joinpoint), in accordance with the reviewer’s comments. Moreover, the periodic fluctuations observed in the disease trend could reflect the impact of intermittent health programs, environmental crises, climatic variations, or economic instabilities [35, 60–62].

These interpretations should be considered in light of the study’s inherent limitations, including its ecological design, the lack of age- or region-specific data, and the omission of important confounding factors such as climatic conditions, livestock practices, and access to healthcare services. These limitations, as well as suggested methodological improvements and future research directions, are further elaborated in the Limitations and Recommendations sections.

## Conclusion

This ecological study revealed a moderate inverse relationship between the Human Development Index (HDI) and the incidence of human brucellosis (HB) in Iran over a 23-year period, alongside a slight upward trend in national HB rates. While this association may reflect the benefits of development—such as stronger health systems and improved disease control—it should be interpreted with caution due to the study’s population-level design. Nonetheless, the findings emphasize the potential value of integrating socio-developmental indicators into zoonotic disease control strategies and provide a foundation for more granular, multilevel, and One Health–oriented research in the future.

## Limitations

Several limitations must be acknowledged. The ecological design of the study prevents inference at the individual level and raises the risk of ecological fallacy. The absence of disaggregated data—by region, age, or sex—limited the ability to conduct more nuanced analyses. Underreporting, variability in case definitions, and potential changes in diagnostic capacity over time may have introduced systematic biases. Additionally, the regression models had relatively low explanatory power and did not account for important confounders such as climate variability, livestock practices, and healthcare access.

## Recommendations

Despite these limitations, this work offers an initial step toward understanding the macro-structural determinants of human brucellosis in the region. Future studies should incorporate multi-level and spatial models, integrate climatic and veterinary data, and explore the role of One Health frameworks to better guide policy and intervention strategies.

## Supplementary Information

Supplementary Material 1.

## Data Availability

All data generated or analyzed during this study are included in this published article and its supplementary information files. Human brucellosis incidence data were obtained from the official repository of the Iranian Center for Communicable Diseases Control, Ministry of Health and Medical Education (available at: [https://icdc.behdasht.gov.ir/brucellosis_situation](https://icdc.behdasht.gov.ir/brucellosis_situation)). The Human Development Index (HDI) data were retrieved from the United Nations Development Programme Human Development Data Center (available at: [https://hdr.undp.org/data-center/specific-country-data#/countries/IRN] (https://hdr.undp.org/data-center/specific-country-data#/countries/IRN)). To ensure long-term accessibility, the extracted datasets have also been provided as supplementary files accompanying this article.
